# The Role of Inspiratory Muscle Strength in Functional Capacity and Left Atrial Strain in Patients With Heart Failure With Mildly Reduced and Preserved Ejection Fraction

**DOI:** 10.1002/clc.70261

**Published:** 2026-04-01

**Authors:** Huu Dat Tran, Thi Ha Le, Thi Hoai Thu Bui, Duc Dung Doan, Thi Kim Lien Nguyen

**Affiliations:** ^1^ Department of Rehabilitation Vinmec Smart City International Hospital Hanoi Vietnam; ^2^ Department of Rehabilitation Haiduong Medical Technical University Haiduong Vietnam; ^3^ Center of Rehabilitation Bach Mai Hospital Hanoi Vietnam; ^4^ Department of Cardiology Vinmec Times City International Hospital Hanoi Vietnam; ^5^ Department of Rehabilitation Hanoi Medical University Hanoi Vietnam; ^6^ Department of Rehabilitation Vietduc University Hospital Hanoi Vietnam

**Keywords:** exercise capacity, heart failure, inspiratory muscle strength, left atrial strain reservoir

## Abstract

**Objectives:**

This study aimed to explore the correlation among inspiratory muscle strength, left atrial strain reservoir (LAsr), and functional capacity in patients with heart failure with preserved and mildly reduced ejection fractions (HFpEF and HFmrEF).

**Methods:**

This cross‐sectional study enrolled 40 patients with HFpEF and HFmrEF. Regression analyses were conducted at both univariate and multivariate levels to explore the relationships between maximal inspiratory pressure (MIP), peak oxygen consumption (pVO_2_), six‐minute walk distance (6MWD), and LAsr.

**Results:**

MIP significantly correlated with pVO_2_ (*r* = 0.400, *p* = 0.011) and 6MWD (*r* = 0.549, *p* < 0.001) in univariate analyses, with multivariable regression confirming its independent association with pVO_2_ (*β* = 0.07, 95% CI: 0.02–0.14, *p* = 0.044) and 6MWD (*β* = 1.14, 95% CI: 0.04–2.23, *p* = 0.043). While univariate analysis showed a meaningful correlation between MIP and LArs (*r* = 0.364, *p* = 0.021), this was not notable in the multivariable model (*β* = 0.09, 95% CI: −0.05–0.24, *p* = 0.181).

**Conclusions:**

MIP is a significant predictor of exercise capacity in HFpEF and HFmrEF; however, its relationship with LAsr was influenced by confounding factors. It is essential to address inspiratory muscle weakness for effective management of patients with HFpEF and HFmrEF.

**Trial Registration:**

This trial was registered in the Thai Clinical Trials Registry (https://www.thaiclinicaltrials.org/) under the trial ID TCTR20231121001.

## Graphical abstract

In patients with HFpEF and HFmrEF, maximal inspiratory pressure is independently associated with exercise capacity, including peak oxygen uptake and 6‐minute walk distance, as well as left atrial reservoir strain. These findings highlight inspiratory muscle strength as a relevant functional determinant of exercise intolerance, independent of age and conventional echocardiographic measures of cardiac function.

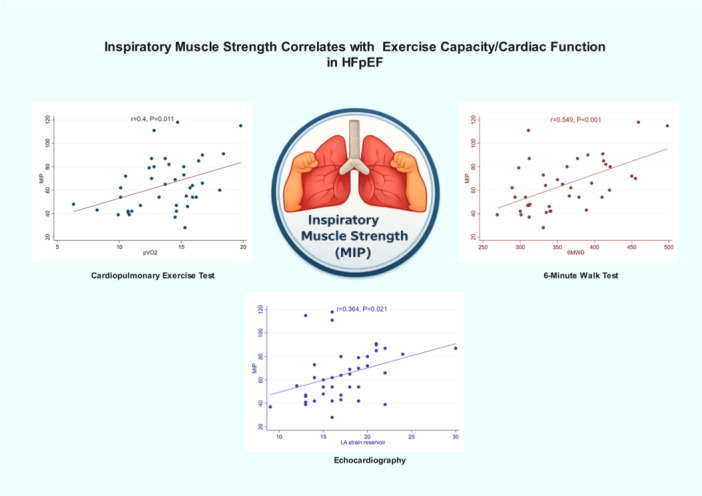

## 1 Introduction

Heart failure (HF) is a clinical syndrome characterized by breathlessness, ankle edema, fatigue, and potential accompanying signs such as increased jugular venous pressure, crackling sounds in the lungs, and edema in the extremities [1]. It is caused by anomalies in heart structure and/or function, resulting in increased intracardiac pressure and/or diminished cardiac output during relaxation or exercise [1]. This condition leads to impairment in exercise performance and quality of life, increasing comorbidity, mortality rates, and healthcare costs [2, 3]. The prevalence of HF in major reviews has been reported to range from 1.3% to 6.7%, with an incidence of 0.5−1.7 cases per 1000 person‐years in several Asian countries, and these numbers are continuing to rise [4, 5]. Among the different phenotypes of HF, HF with mildly reduced and preserved ejection fractions (HFmrEF and HFpEF) accounts for over 50% of all HF cases and is associated with high mortality [1, 6, 7]. Most patients with HFpEF have comorbidities, a sedentary lifestyle, and exercise intolerance [8, 9]. Inspiratory muscle weakness (IMW) is prevalent in HFpEF, with a reported prevalence of 27.5%−42% [10, 11, 12]. It may exacerbate dyspnea and exercise intolerance by impairing respiratory mechanics [13].

Maximal inspiratory pressure (MIP) is a key parameter for assessing inspiratory muscle strength and has been linked to functional outcomes such as exercise intolerance and quality of life in patients with HF [14]. Additionally, peak oxygen consumption (pVO_2_) assessed by cardiopulmonary exercise test (CPET) and left atrial (LA) strain reservoir are becoming popular and reliable prognostic factors for HF outcomes. While MIP is readily measured and available in many hospitals and clinics, pVO_2_ and LA strain reservoir are more complex to assess and less commonly available in many facilities. Despite its clinical significance, the correlation between MIP and pVO_2_ is still controversial [12, 14] and that between MIP and LA strain reservoir remains unclear. Establishing a clear link between MIP, functional capacity, and LA strain reservoir could support interventions aimed at enhancing MIP, thereby improving exercise tolerance and overall outcomes.

This study investigated the correlation among MIP, LA strain reservoir, and exercise capacity in patients with HFpEF and HFmrEF. We hypothesized that MIP is independently associated with both exercise capacity and LA strain reservoir, highlighting its potential role as a therapeutic target in HF management.

## 2 Methods

### 2.1 Participants

This cross‐sectional observational study was conducted using baseline data from a prospective study of patients with HFpEF and HFmrEF. Forty patients diagnosed with HFpEF and HFmrEF from the Outpatient Clinic of the Cardiology Center were enrolled between September 2024 and January 2025. A physiatrist screened and evaluated all participants. Eligible patients were as follows: (1) adults aged 18 years or older with chronic HF symptoms and left ventricular ejection fraction (LVEF) > 40%, (2) with either N‐terminal pro B‐type natriuretic peptide (NT‐proBNP) > 220 pg/mL or elevated left ventricular filling pressure (at rest or exercise) on echocardiography, (3) catergorized as New York Heart Association (NYHA) class II−III, and (4) stable HF medications for at least 4 weeks. Patients were excluded if they: (1) could not perform baseline exercise tests, (2) required intravenous therapies or mechanical support within a week, or had acute decompensated HF, (3) had recent angina or surgery (within 3 months), (4) had end‐stage renal disease or prior heart transplantation, or (5) declined to participate in the study.

### 2.2 Outcome Variables

Data on age, sex, body mass index (BMI), NYHA functional class, HF phenotypes, NT‐proBNP, echocardiographic parameters (LVEF, LV cavity, E/e’ medial, E/e’ average, LA strain reservoir), comorbidities, and medications were collected from electronic medical records. Exercise capacity (6‐minute walk distance [6MWD], pVO_2_, anaerobic threshold, and respiratory exchange ratio) and MIP were evaluated by an experienced physician at baseline, within a predefined time window of the same day or within 48 h, during a clinically stable condition, before any intervention.

### 2.3 Outcome Measurements

#### 2.3.1 Inspiratory Muscle Strength

The MIP was assessed using a digital handheld device (Contec Respiratory Pressure Meter, RPM10) in a standing position with unrestricted thoracic movement. Measurements began at the start of inhalation after exhaling to the residual volume, followed by forceful inhalation sustained for 2–3 s. A rest period of 30–60 s was required between attempts. To ensure accuracy, the differences between consecutive measurements should not exceed 10%, with at least three satisfactory attempts recorded and the highest value reported [15]. An IMW is generally defined as an MIP of < 70% of the predicted value [16].

#### 2.3.2 CPET

A CPET system equipped with a cycle ergometer (Geratherm Respiratory GmbH) was used. The test began with a short warm‐up period, followed by a ramp increase in workload at 25 W/min, gradually approaching the patient's maximum exercise capacity. The participants were instructed to hold a consistent pedaling cadence of 55–65 revolutions per minute. The test was continued until the patient experienced voluntary exhaustion or met clinical termination criteria. A cool‐down period followed to ensure safe recovery. The pVO_2_ was recorded after the test was completed [17].

#### 2.3.3 Six‐Minute Walk Test (6MWT)

The test was performed following the protocol supported by the American Thoracic Society guidelines [18]. Patients were required to walk continuously in a closed 30 m linear corridor, aiming to cover as much distance as possible within 6 min. The distance walked (6MWD) served as a measure of exercise tolerance, with greater distances indicating greater exercise capacity.

#### 2.3.4 Echocardiography

Echocardiographic parameters were analyzed using a single piece of equipment (Vivid S70, GE Healthcare) by a qualified cardiologist, in accordance with the guidelines of the American Society of Echocardiography [19]. Left ventricular cavity dimensions were measured at end‐diastole, following standard guideline‐based echocardiographic protocols. The tissue Doppler imaging of septal and lateral mitral annulus was obtained in apical four‐chamber view, including the analysis of mitral e’ and mitral E/e’ estimating LV filling pressure. All measurements were analyzed and averaged in three to five consecutive beats by a well‐trained cardiologist. An average E/e′ ratio > 14 was considered indicative of elevated LV filling pressure [19]. LA strain reservoir was measured using two‐dimensional speckle‐tracking echocardiography from apical four‐ and two‐chamber views, with endocardial border tracing performed at ventricular end‐systole. The zero‐reference point was set at LV end‐diastole, and the LA strain reservoir was defined as the peak positive longitudinal strain during ventricular systole.

#### 2.3.5 Statistical Analysis

Categorical variables are summarized as percentages, while continuous variables are presented as means ± standard deviation. The normality of the data was assessed using the Shapiro−Wilk test. Comparisons between the two groups (with and without IMW) for continuous variables were performed using an independent *t*‐test. Categorical variables were analyzed using the chi‐squared test or Fisher's exact test. Univariate regression was performed using Pearson's correlation coefficient. Multivariate regression analyses were conducted to determine the correlation between MIP and exercise capacity after adjusting for age, sex, NYHA functional class, LVEF, BMI, NT‐proBNP, diabetes, atrial fibrillation, β‐blockers, and E/e’ medial, as well as the correlation between MIP and LA strain reservoir after adjusting for age, sex, LVEF, BMI, diabetes, atrial fibrillation, medications, and E/e’ medial. All analyses were performed using Stata 17.0 (StataCorp LP, College Station, TX, USA), with statistical significance set at *p* < 0.05.

## 3 Results

The study included 40 patients with a mean age of 67.7 ± 8.9 years, predominantly male (62.5%). The mean NT‐proBNP level was 543.2 ± 243.6 pg/mL, with 20% of patients classified as NYHA functional class III. The average peak VO_2_ was 13.7 ± 3.6 mL/kg/min, and the mean LA strain reservoir was 17.3 ± 3.9%. Most atrial fibrillation cases in our cohort were classified as persistent atrial fibrillation, with no patients meeting criteria for paroxysmal atrial fibrillation. Inspiratory muscle strength, measured by MIP, was 64.5 ± 22.2 cmH_2_O. The other characteristics are represented in Table 1.

**Table 1:** Participants' characteristics.

	Overall (*n* = 40)	No IMW (*n* = 26)	IMW (*n* = 14)	*p* value
Age (years)	67.7 ± 8.9	66.8 ± 8.7	69.4 ± 9.3	0.402
Male/female	25/15	18/8	7/7	0.231
Body mass index (kg/m^2^)	25.3 ± 2.3	25.6 ± 2.5	24.7 ± 2.0	0.237
NYHA functional class II/III, *n*	32/8	23/3	9/5	0.068
HFpEF/HFmrEF	27/13	14/12	13/1	0.012*
NT‐proBNP (pg/mL)	543.2 ± 243.6	489.3 ± 197.2	597 ± 277.1	0.164
Echo				
LV cavity (mm)	47.2 ± 7.1	48.5 ± 8.2	44.8 ± 3.8	0.132
LVEF (%)	55.1 ± 8.9	54.1 ± 9.5	57.0 ± 7.8	0.328
E/e’ medial	12.6 ± 3.3	12.7 ± 3.4	12.5 ± 3.2	0.892
E/e'av	11.1 ± 3.3	11.5 ± 3.8	10.4 ± 2.1	0.375
LA strain reservoir (%)	17.3 ± 3.9	18.2 ± 4.0	15.6 ± 3.2	0.044*
Comorbidities, *n* (%)				
Hypertension	27 (67.5)	17 (65.4)	10 (71.4)	0.697
Diabetes	20 (50)	13 (50)	7 (50)	1
Atrial fibrillation	15 (37.5)	8 (30.8)	7(50)	0.231
Exercise capacity				
pVO_2_ (mL/kg/min)	13.7 ± 3.6	15.3 ± 2.2	12.5 ± 3.6	0.045*
AT (%)	8.5 ± 1.9	8.9 ± 1.7	7.7 ± 2.0	0.059
RER	1.096 ± 0.024	1.097 ± 0.026	1.095 ± 0.021	0.811
6MWD (m)	359.1 ± 55.0	373.9 ± 49.5	331.6 ± 55.7	0.018*
MIP (cmH_2_O)	64.5 ± 22.2	73.2 ± 18.1	48.3 ± 20.3	< 0.001*
Medication, *n* (%)				
Beta‐blockers	37 (92.5)	23 (88.5)	14 (100)	0.186
Diuretics	35 (87.5)	22 (84.6)	13 (92.9)	0.452
ACEIs/ARBs/ARNIs	35 (87.5)	22 (84.6)	13 (92.9)	0.452
MRAs	11 (27.5)	11 (42.3)	0 (0)	0.004*
SGLT2i	36 (90)	23 (88.5)	13 (92.9)	0.658

Abbreviations: 6MWD, 6‐minute walk distance; ACEI, angiotensin‐converting enzyme inhibitor; ARB, angiotensin receptor blocker; ARNI, angiotensin receptor‐neprilysin inhibition; AT, anaerobic threshold; av, average; HF‐mrEF, heart failure with mid‐range ejection fraction; HF‐pEF, heart failure with preserved ejection fraction; IMW, inspiratory muscle weakness; LV, left ventricular; LVEF, left ventricular ejection fraction; MIP, maximal inspiratory pressure; MRA, mineralocorticoid receptor antagonists; NT‐proBNP, N‐terminal pro B‐type natriuretic peptide; NYHA, New York Heart Association; pVO_2_, peak oxygen uptake; RER, respiratory exchange ratio; SGLT2i, sodium‐glucose cotransporter 2 inhibitor.

* *p* < 0.05.

Patients with IMW (*n* = 14) differed significantly from those without IMW (no IMW, *n* = 26) in several key parameters. While age, BMI, NT‐proBNP levels, and echocardiographic measures such as LVEF and E/e’ ratios were similar between the groups, the IMW group had a significantly lower LA strain reservoir (15.6 ± 3.2 vs. 18.2 ± 4.0, *p* = 0.044). Exercise capacity was notably reduced in the IMW group, with lower peak VO_2_ (12.5 ± 3.6 vs. 15.3 ± 2.2 mL/kg/min, *p* = 0.045) and 6MWD (331.6 ± 55.7 vs. 373.9 ± 49.5 m, *p* = 0.018). Additionally, the IMW group showed a distinct distribution of HF phenotypes (HFpEF vs. HFmrEF, *p* = 0.012) and no MRA use compared with 42.3% in the no IMW group (*p* = 0.004) (Table 1).

**Figure 1:**
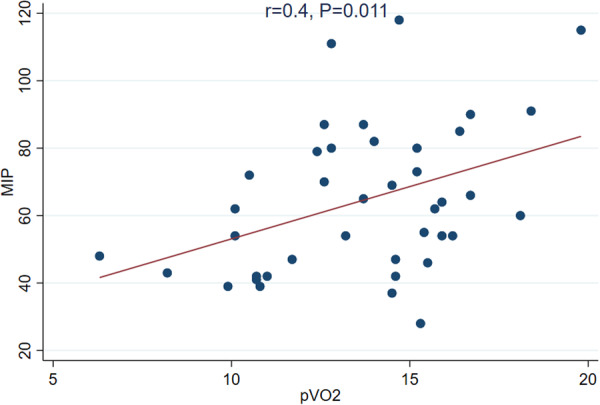
The scatter plot illustrates the positive relationship between MIP and pVO₂, highlighting the role of inspiratory muscle strength in cardiopulmonary performance.

Univariate correlation analysis revealed significant correlations between MIP and several variables. Positive correlations were observed between pVO_2_ (*r* = 0.400, *p* = 0.011), 6MWD (*r* = 0.549, *p* < 0.001), and LA strain reservoir (*r* = 0.364, *p* = 0.021). A notable negative correlation was found between MIP and age (*r* = −0.410, *p* = 0.009). Conversely, no meaningful associations were observed between MIP and LVEF (*r* = 0.159, *p* = 0.327), NT‐proBNP (*r* = −0.276, *p* = 0.085), or BMI (*r* = −0.017, *p* = 0.919) (Table 2 and Supporting Information Figures) (Figure 1).

**Table 2:** Univariable correlation between MIP and other outcomes.

Variable	Correlation coefficient	*p* value
pVO_2_	0.400	0.011*
6MWD	0.549	< 0.001*
LA strain reservoir	0.364	0.021*
Age	−0.410	0.009*
LVEF	0.159	0.327
NT‐proBNP	−0.276	0.085
BMI	−0.017	0.919

Abbreviations: 6MWD, 6‐minute walk distance; BMI, body mass index; LA, left atrial; LVEF, left ventricular ejection fraction; NT‐proBNP, N‐terminal pro B‐type natriuretic peptide; pVO_2_, peak oxygen uptake.

* *p* < 0.05.

In multivariable linear regression analysis, MIP demonstrated significant positive correlations with key measures of exercise capacity, including pVO_2_ (*β* = 0.07, 95% CI: 0.02 to 0.14, *p* = 0.044) and 6MWD (*β* = 1.14, 95% CI: 0.04 to 2.23, *p* = 0.043). However, the relationship between MIP and LA strain reservoir (*β* = 0.09, 95% CI: −0.05 to 0.24, *p* = 0.181) was not statistically significant. All covariates in the regression model are presented in Table 3.

**Table 3:** Multivariable regression analysis for correlation between MIP and pVO_2_, 6MWD, or LA strain reservoir.

	pVO_2_			6MWD			LA strain reservoir		
	*β*‐coefficient	95% CI	*p* value	*β*‐coefficient	95% CI	*p* value	*β*‐coefficient	95% CI	*p* value
Age (year)	−0.008	−0.17 to 0.16	0.918	1.04	−1.59 to 3.67	0.415	−0.06	−0.40 to 0.28	0.712
Male	0.68	−2.52 to 3.88	0.659	4.46	−46.12 to 55.03	0.855	−0.50	−6.79 to 5.79	0.867
NYHA class III	0.06	−2.69 to 2.81	0.963	7.96	−35.58 to 51.49	0.705			
MIP (cmH_2_O)	0.07	0.02 to 0.14	0.044*	1.14	0.04 to 2.23	0.043*	0.09	−0.05 to 0.24	0.181
LVEF (%)	−0.07	−0.24 to 0.09	0.362	−0.52	03.16 to 2.13	0.685	−0.11	−0.49 to 0.28	0568
E/e’ medial	−0.59	−1.04 to −0.14	0.014*	−4.51	−11.69 to 2.66	0.202	−0.43	−1.37 to 0.52	0.347
Diabetes	3.00	−0.17 to 6.17	0.062	−0.39	−50.51 to 49.73	0.987	0.79	−4.80 to 6.39	0.766
Atrial fibrillation	1.69	−1.35 to 4.73	0.256	−25.31	−73.37 to 22.74	0.282	−1.07	−6.37 to 4.24	0.673
Beta‐blockers	3.02	−1.15 to 7.18	0.145	−51.83	−117.65 to 13.99	0.115	0.76	−7.75 to 9.27	0.851
Diuretics							−2.56	−12.84 to 7.71	0.301
ACEIs/ARBs/ARNIs							2.02	−4.97 to 9.01	0.545
MRAs							−3.63	−10.89 to 3.63	0.301
BMI (kg/m^2^)	−0.23	−0.80 to −0.34	0.400	0.51	−8.48 to 9.50	0.906	0.28	−7.75 to 9.27	0.570
NT‐proBNP (pg/mL)	0.003	−0.007 to 0.012	0.553	0.06	−0.10 to 0.21	0.475			
Adjusted *R* ^2^	0.20			0.38			0.21		

Abbreviations: 6MWD, 6‐minute walk distance; ACEI, angiotensin‐converting enzyme inhibitor; ARB, angiotensin receptor blocker; ARNI, angiotensin receptor‐neprilysin inhibition; BMI, body mass index; LA, left atrial; LVEF, left ventricular ejection fraction; MIP, maximal inspiratory pressure; MRA, mineralocorticoid receptor antagonist; NT‐proBNP, N‐terminal pro B‐type natriuretic peptide; NYHA, New York Heart Association; pVO_2_, peak oxygen uptake; SGLT2i, sodium‐glucose cotransporter 2 inhibitor.

* *p* < 0.05.

## 4 Discussion

**Figure 2:**
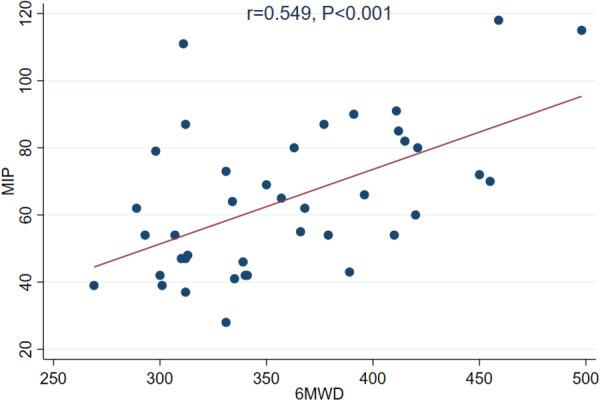
This figure demonstrates the association between inspiratory muscle strength and functional exercise capacity, as indicated by 6MWD.

The findings from this observational study indicate that (1) MIP is a significant and independent correlate of exercise capacity in patients with HFpEF and HfmrEF, and (2) MIP is not associated with the LA strain reservoir after adjusting for other confounders. This preliminary study evaluated the correlation between inspiratory muscle strength and the LA strain reservoir, an important prognostic factor in patients with HFpEF (Figure 2).

**Figure 3:**
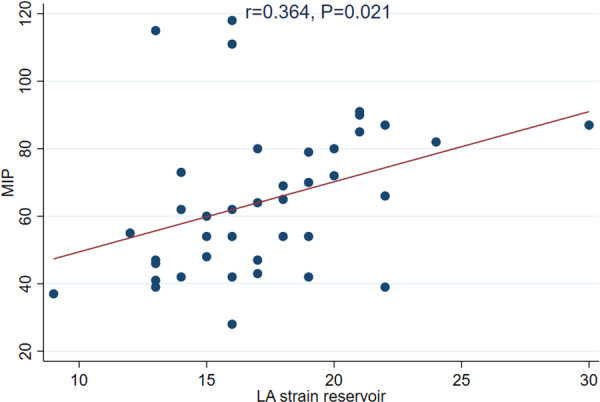
The data depict the relationship between MIP and LA strain reservoir, suggesting potential implications for cardiac mechanics and inspiratory muscle strength.

This study demonstrates a strong positive correlation between MIP and 6MWD in HFpEF patients (*r* = 0.549, *p* < 0.001). Multivariable analysis further confirmed this relationship (*β* = 1.14, 95% CI: 0.04 to 2.23, *p* = 0.043), underscoring the role of respiratory muscle strength in determining walking performance. As the 6MWD is the most common submaximal exercise test, the ability of MIP to predict the 6MWD highlights its clinical relevance. These results align with previous findings [14, 20, 21], suggesting that interventions targeting inspiratory muscle strength, such as inspiratory muscle training (IMT), may enhance the 6MWD and improve functional outcomes in patients with HFpEF (Figure 3).

The pVO_2_ is considered a standard measure of functional capacity in cardiovascular diseases and a key prognostic indicator in patients with HF [22, 23, 24, 25]. This indicates an individual's capacity to supply and consume oxygen at maximum exertion. The pVO_2_ reflects an individual's fitness level and endurance tolerance [26]. The pVO_2_ is a crucial assessment of patients with HF including diagnosis, treatment planning, monitoring, and prognosis. Some previous studies reported a positive correlation between MIP and pVO_2_ [14, 20, 21]. For instance, most recently in 2024, Shah et al. showed a notable correlation of MIP and pVO_2_ in univariable regression (*r* = 0.430, *p* = 0.0019) and multivariable regression (*β* = 0.06, *p* = 0.07) in patients with HF [14]. The present study aligns with previous observational trials demonstrating significant correlations through both univariate regression (*r* = 0.400, *p* = 0.011) and multivariate regression (*β* = 0.07, 95% CI: 0.02 to 0.14, *p* = 0.044), respectively. Interestingly, Palau et al. reported no notable correlation between MIP and pVO_2_ in patients with HFpEF on either univariate or multivariate analyses [12]. The authors attributed this finding to differences between the two populations being evaluated (HFpEF vs. all HF phenotypes). In the present study, we provide further evidence that MIP is an independent predictor of functional capacity in patients with HFpEF.

The LA strain reservoir is a measure of atrium's function, reflecting its ability to expand and store blood during left ventricular systole (when the ventricle is contracting and ejecting blood). It is derived using speckle‐tracking echocardiography and represents the deformation of the LA wall [27]. Recently, the LA strain reservoir has emerged as a reliable prognostic marker, with higher values related to better outcomes in patients with HFpEF, including reduced risk of death and hospitalization [28, 29]. Although direct evidence linking inspiratory muscle strength to the LA strain reservoir is lacking, several mechanisms have been proposed to explain this relationship: (1) stronger inspiratory muscles, reflected by higher MIP, generate more efficient negative intrathoracic pressure, enhance venous return, and lower LA pressure, which improves LA compliance and strain [30, 31, 32] (2) Additionally, improved diaphragmatic efficiency and reduced pulmonary congestion may contribute to better hemodynamics, decrease atrial stiffness, and facilitate LA reservoir expansion during ventricular systole [30]. The present study shows a meaningful correlation between MIP and LA strain reservoir with univariate regression (*r* = 0.364, *p* = 0.021) but not with multivariate regression (*β* = 0.09, 95% CI: −0.05 to 0.24, *p* = 0.181). These results indicate that the correlation could be affected by confounding factors (age, sex, comorbidities, and medications).

Interestingly, age is a well‐recognized determinant of exercise capacity and atrial remodeling [33, 34]; however, it did not emerge as an independent predictor in the multivariable analyses of this study. This finding likely reflects the inclusion of more proximal physiological variables, such as MIP and diastolic function parameters, which may mediate the effects of aging on cardiopulmonary performance. In addition, the relatively homogeneous age distribution of this HFpEF cohort may have limited the ability to detect an independent age effect. Similarly, although atrial fibrillation has a substantial impact on LA function [35], most patients in this study had persistent atrial fibrillation, resulting in a relatively homogeneous rhythm status across the cohort. Consequently, the effect of atrial fibrillation on LA strain was likely uniform and did not contribute meaningfully to between‐patient variability, thereby limiting its ability to emerge as an independent determinant in a multivariable model.

This study has some limitations that need to be considered: (1) the relatively small sample size limits the ability to detect significant effects, especially regarding the correlation between MIP and LA strain reservoir, (2) the population focused on HFpEF and HFmrEF and cannot be directly extrapolated to all patients with HF, and (3) the lack of LAVI assessment limits interpretation of atrial remodeling, particularly in patients with atrial fibrillation, in whom atrial size may provide complementary prognostic information. Further research is required to delineate the mechanisms linking MIP and LA strain reservoir and to evaluate the impact of IMT on these parameters.

## 5 Conclusion

This study provides evidence that inspiratory muscle strength, as measured using MIP, is an independent predictor of exercise capacity in patients with HFpEF and HFmrEF. However, the relationship between MIP and LA strain reservoir remains unclear. These findings emphasize the need for targeted interventions to improve inspiratory muscle strength as a means of enhancing exercise tolerance and overall outcomes in such a patient population.

## Author Contributions

Tran Huu Dat conceptualized the study. Tran Huu Dat, Ha Le Thi, Bui Thi Hoai Thu, Dung Doan Duc, and Lien Nguyen Thi Kim designed and contributed to the methodology. Tran Huu Dat and Bui Thi Hoai Thu collected research data. Tran Huu Dat performed the data analysis. Tran Huu Dat and Tran Huu Dat drafted the initial version of the manuscript. All five authors critically reviewed and approved the final manuscript as submitted.

## Funding

The authors received no specific funding for this work.

## Ethics Statement

The study was approved by the Ethics Review Committee of Vinmec Healthcare System (Number: 102/2024) and the Institutional Review Board of the Faculty of Medicine, Chulalongkorn University (Number: 0790/66). Written informed consent was obtained from all participants prior to their enrollment in the study.

## Conflicts of Interest

The authors declare no conflicts of interest.

## Supporting information


**Figure 1:** Correlation between MIP and pVO2. **Figure 2:** Correlation between MIP and 6MWD. **Figure 3:** Correlation between MIP and LA strain reservoir.

## Data Availability

The data supporting the findings of this study are available from the corresponding author upon reasonable request.
