# Supplementary material for: The Role of Inspiratory Muscle Strength in Functional Capacity and Left Atrial Strain in Patients With Heart Failure With Mildly Reduced and Preserved Ejection Fraction
Source: Clin Cardiol. 2026 Apr 1;49(4):e70261. doi: 10.1002/clc.70261 (PMC13042886; doi:10.1002/clc.70261)
Supplement: Supplementary file 1 — Figure 1: Correlation between MIP and pVO2. Figure 2: Correlation between MIP and 6MWD. Figure 3: Correlation between MIP and LA strain reservoir. [file CLC-49-e70261-s001.docx]

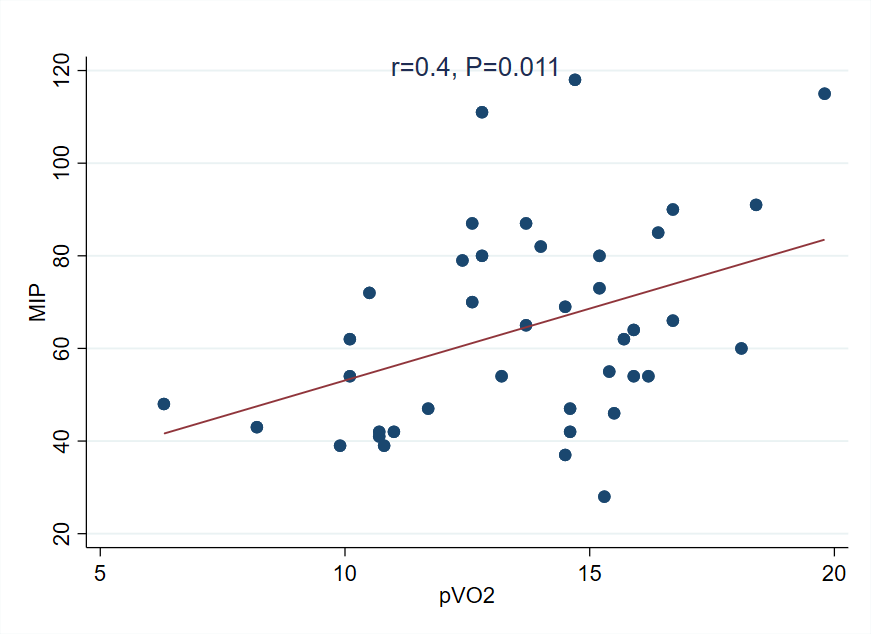


**Figure 1**. Correlation between MIP and pVO2

MIP – maximal inspiratory pressure; pVO_2_ – maximal oxygen uptake


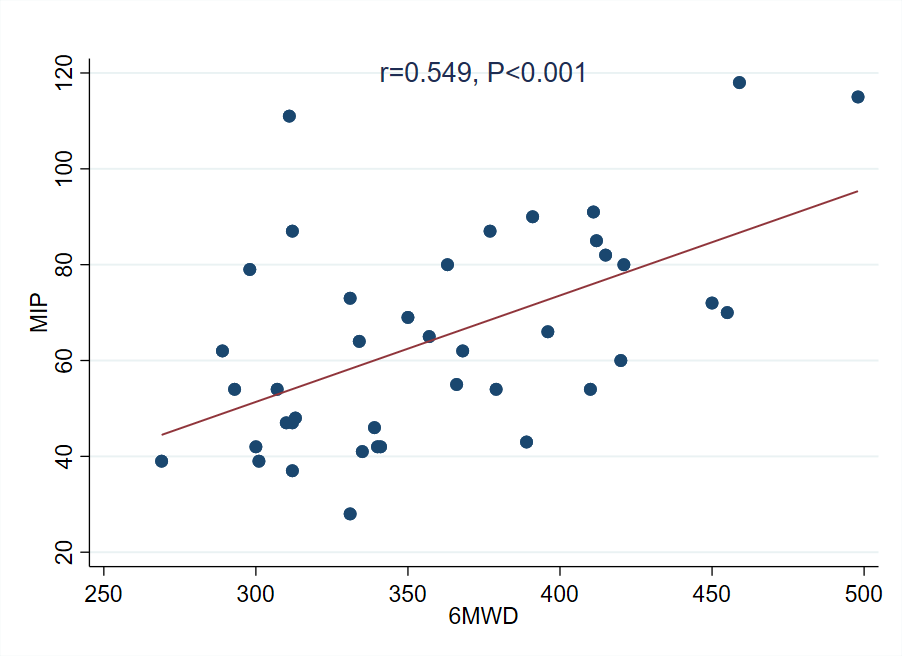


**Figure 2**. Correlation between MIP and 6MWD

6MWD – 6-minute walk distance; MIP – maximal inspiratory pressure


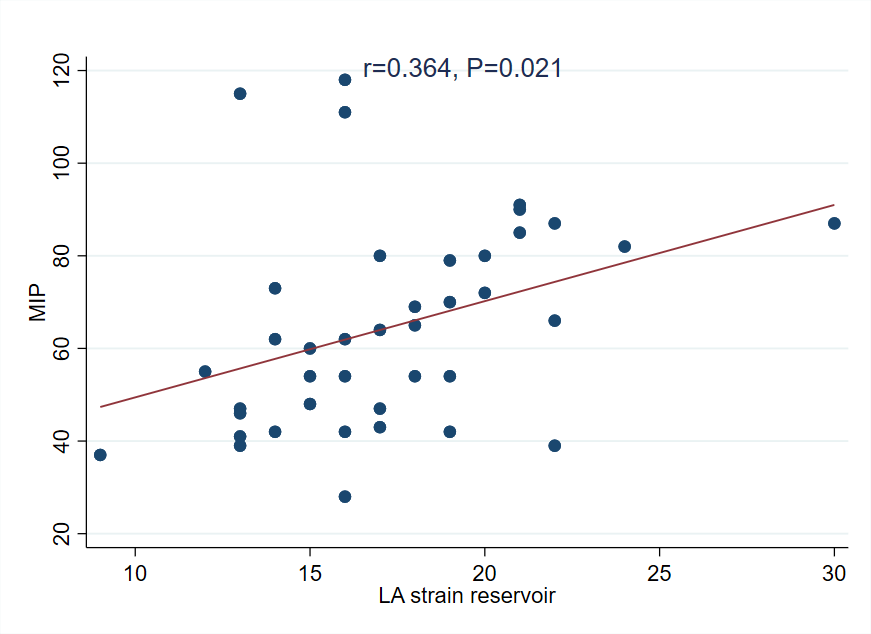


**Figure 3**.Correlation between MIP and LA strain reservoir

LA – left atrial; MIP – maximal inspiratory pressure
